# Optimization of the Formulation of Rice Biscuits Supplemented with *D. Edulis* (L.) Powder Using Response Surface Methodology

**DOI:** 10.1155/2021/5215367

**Published:** 2021-08-31

**Authors:** Eliane Flore Eyenga, Hippolyte Tene Mouafo, Mercy Bih Loh Achu, Wilfred F. Mbacham, Sali Atanga Ndindeng

**Affiliations:** ^1^Laboratory of Food Science and Technology, Institute of Agricultural Research for Development (IRAD), P.O. Box 2123 Yaoundé, Cameroon; ^2^Department of Biochemistry, Faculty of Science, University of Yaoundé 1, P.O. Box 812, Yaoundé, Cameroon; ^3^Centre for Food and Nutrition Research, Institute of Medical Research and Medicinal Plant Studies, P.O. Box 13033, Yaoundé, Cameroon; ^4^Africa Rice Center (AfricaRice), M'be Research Station, P.O. Box 2551, Bouake, Ivory Coast, South Africa

## Abstract

Trends in the food industry are nowadays directed towards the reduction of the level of trans fatty acids in food intended for human consumption. The present study was designed and aimed at valorizing *Dacryodes edulis* (L.) powder as a substitute for margarine in the production of functional rice biscuits. The effect of substituting margarine and refined wheat flour with *D. edulis* powder locally called safou and rice flour, respectively, at different proportions was assessed for the sensory and physicochemical properties of the formulated biscuits. For this, statistical models were developed, validated, and optimized using the response surface methodology with the Doehlert design as a tool. The results showed that an increase in the substitution rate of margarine with of *D. edulis* powder enhanced the aroma while the substitution of refined wheat flour with rice flour led to an improvement of the overall quality of the biscuits. The optimal composition of dough for the production of biscuits with satisfying sensory properties was 20.24% of wheat flour, 24.51% of rice flour, 19.09% of margarine, and 2.47% of *D. edulis* powder. The optimized biscuit which scored the highest overall acceptability contained proteins (10.33 g/100 g DM), fat (27.66 g/100 g DM), crude fibers (2.5 g/100 g DM), ash (3.55 g/100 g DM), and carbohydrates (54.01 g/100 g DM). It has an energy density of 506.3 ± 0.1 kcal/g and could therefore be suitable for the management of malnutrition. Mineral analysis revealed that the biscuit contained sodium (0.200 mg/100 g), potassium (0.192 mg/100 g), phosphorus (0.123 mg/100 g), iron (33.60 ppm), and zinc (26.81 ppm) at levels satisfying the recommended daily intakes. The results of this study demonstrated the suitability of safou as substitute of margarine in the rice biscuit formulation and suggests the potential of the formulated biscuits in the management of malnutrition and noncommunicable diseases such as hypertension, obesity, and cardiovascular diseases.

## 1. Introduction

Bakery products with a good nutritional profile, sensory characteristics, and texture are nowadays most popular among all age groups and appreciated by consumers [1]. Due to the growing consumer demands for convenient food, biscuits represent one of the fast-growing segments of bakery products. Ready to eat, unique taste, readily available, long shelf life, and good eating quality are characteristics which enhance its consumption [2]. However, most biscuits are made with butter and margarine which contain high amount of fats, with saturated and trans fatty acids. They also contain good quantities of sugar and salt which might be the leading causes of health problems such as diabetes, hypertension, obesity, and cardiovascular diseases [3]. As the prevalence of these noncommunicable diseases increases worldwide, consumers become worried about their health and so demand snacks which can provide health benefits with a good nutritional value.

Wheat flour is a basic ingredient in the preparation of biscuits [4]. However, its conditions of cultivation associated to the high cost of its importation as well as the phenomenon of gluten intolerance have led to the development of several researches in order to find alternatives to wheat. Nowadays, researches are directed towards the use of local raw materials due to their composition which will improve the nutritional composition of biscuits and also to their great potential for improving the sustainability of food systems [5]. In this light, the promotion of the use of composite flours, which integrate flours derived from local crops (cereals, tubers, roots, and leguminous seeds), in bakery products such as biscuits is being encouraged. Studies have reported the total or partial substitution of wheat flour in bread, cake, and biscuit preparations [6]. Moreover, Vitali et al. [7] showed that the use of composite flour improves the nutritional value of the bakery products. The authors also noticed that when rice and fruits are used in the formulation of composite flour, the nutritional value of the bakery product is significantly improved. In fact, rice is free from gluten and it contains carbohydrates, proteins, fats, vitamins such as thiamine, riboflavin and niacin, and vitamin E [8]. Rice also contains resistant starch for which positive health effects have been demonstrated [9, 10].

Besides rice, other local ingredients for which a growing attention is paid nowadays in biscuit formulation are those rich in health-promoting compounds. Pasqualone et al. [5] demonstrated the use of almond skins rich in bioactive compounds in the formulation of nutritious and functional biscuits. Safou (*D. edulis* (L.)) a highly perishable local food [11] stands as an example of those ingredients which can be valorized in the preparation of functional biscuits for improving its sustainability. Safou is available in Cameroon and contains on the dry weight basis 33–65% of lipids, 15–30% of proteins, 2–5% of ash [12], and several bioactive compounds [13, 14]. Safou pulp is rich in saturated fatty acids and several studies have highlighted their potential for the management of cardiovascular diseases [15]. Hence, in the preparation of biscuits, the substitution of margarine and butter which contain saturated and trans fatty acids with safou pulp appears as an interesting field to explore. Several studies have reported the use of safou pulp in bakery products [16, 17]. In a study recently conducted by Eyenga et al. [14], the nutritional and health-promoting benefits of biscuits made with a composite flour containing wheat, rice, margarine, and safou pulp powder were shown. However, the final quality and acceptability of the biscuits were influenced by the quantity of each ingredient in the formulation. Hence, in order to find out the optimum quantity of each ingredient leading to a satisfying product, the use of the statistical model is of great importance. The most accepted methodology for this kind of studies is the response surface methodology (RSM). It is a statistical tool which is suitably used in the development and optimization of food products [18]. It systematically determines the effects of multiple variables in a mixture on quality attributes while minimizing the number of experiments that must be conducted [19]. The objective of this study is therefore to (i) determine optimum conditions of the substitution of margarine with safou leading to biscuits with good granulometry, crispness, hardness, flavor, and overall quality and (ii) assess the nutritional value of the optimized biscuits.

## 2. Materials and Methods

### 2.1. Sample Collection

**Figure 1:**
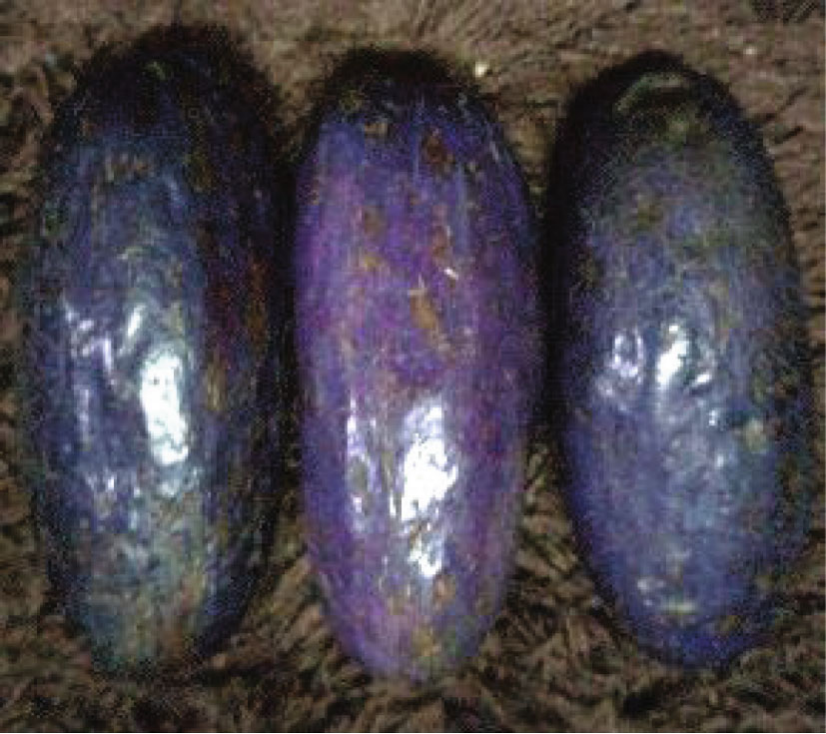
Mature sour fruits of safou (*D. edulis* L.).

Mature sour fruits of *D. edulis* (4 months) were harvested at the experimental farm of the Institute of Agricultural Research for Development (IRAD), located at Nkolbisson in the city of Yaoundé, Centre Region of Cameroon. The safou fruits were blue in colour, with a length and midpoint diameter of 10.0 cm and 5.4 cm, respectively.  Figure 1 illustrates safou fruits.

Fine broken samples of rice, of TOX-3, and 145-TOC-38-2-3 varieties were collected from millers. These two varieties were chosen because they are mainly cultivated in the Ndop Rice Development Hub (rice hub), North West and West Regions of Cameroon [20].

### 2.2. Fabrication of Safou Powder

The method described by Eyenga et al. [14] was used to prepare powder from raw safou fruits. Briefly, the fruits were sorted, washed twice with distilled water, and cut into two halves using a stainless steel knife. Seeds and pericarp were removed, and the fruits were sliced into 1.5 cm^2^ and dried in a Heraeus® D-6450 Hanau drying oven for 24 h at 55–60°C. The chips were removed, cooled at room temperature, crushed using a grinder, and sieved (1 mm mesh sizes). The powder obtained was packaged into plastic bags and stored at 4°C until use. The chemical composition of safou powder is presented in Table 1.

### 2.3. Fabrication of Rice Flour

Rice flour was prepared following the method of Eyenga et al. [14]. Samples of rice was winnowed, washed, and soaked in distilled water for 8 h at room temperature (25 ± 2°C). The rice was removed from water and dried to a moisture content of 20%. Then, the rice was crushed in a regular corn mill (dry milling) before completely drying the flour to 10%–12% moisture. The dried powder was sieved in order to bring the particle sizes to uniform at 50 *μ*m, and its chemical composition previously determined [14] is consigned in Table 1.

### 2.4. Biscuit Production Process

The method described by Eyenga et al. [14] was used to produce the biscuits. Briefly, margarine (Jadida, Yaoundé, Cameroon) was introduced into a clean bowl and creamed thoroughly with a stainless steel spatula until it becomes soft and smooth. Powdered sugar (SOSUCAM, Mbandjock, Cameroon) was added and creamed to give a homogenous mixture. Safou powder was added to the margarine–sugar mixture and properly mixed to obtain a smooth paste. Rice flour and wheat flour were mixed together with baking powder and salt. This flour–baking powder–salt mixture was added to the margarine–sugar–safou mixture and homogenized with the spatula to form a paste. Egg (Yaoundé, Cameroon) and liquid flavor (Vanilla Foster Clark's, Douala, Cameroon) were then added, and the mixture was stirred to obtain the final biscuit dough. Vanilla was chosen because it is the commonly used flavoring agent in baking products. At the dose used, it does not interfere with product aroma [14]. The dough was then put into the biscuit mold, and the desired biscuit shape was formed on a tray lightly greased with margarine. The trays randomly filled with shaped dough were introduced into an electric oven (Panasonic MOV-212, Japan) set at 160°C and baked for 20–25 min. The light-brown baked biscuits obtained were removed and put in a large tray to cool to room temperature before packaging.

### 2.5. Optimization of the Formulation of Rice Biscuits

#### 2.5.1. Experimental Design

The response surface methodology through a Doehlert design was used to perform the experiments. The independent variables and their ranges of variation were wheat flour 120–1200 g, rice flour 120–1200 g, margarine 60–600 g, and safou powder 60–600 g. The responses measured were hardness, aroma, crispness, granular, and overall acceptability of the biscuits.

#### 2.5.2. Modelling

The independent variables were related to the assessed responses through a second-order polynomial model using the following equation.

(1) $$\begin{array}{c} Y=\beta_{0}+\overset{k}{\underset{j=1}{\sum }}\beta_{j}X_{j}+\overset{k}{\underset{j=1}{\sum }}\beta_{jj}{X_{j}}^{2}+\sum \underset{i<j}{\sum }\beta_{ij}X_{i}X_{j}+\varepsilon . \end{array}$$

#### 2.5.3. Validation of the Model

The indicators used for model validation were coefficient of determination (*R*^2^), the adjusted *R*^2^, the absolute analysis of average deviation (AAD), the bias factor (Bf), the accuracy factor (Af), and the lack of fit. Adequacy was considered when *R*^2^ ≥ 80%, adj − *R*^2^ ≥ 80%, AAD⟶0, 0.75 < Af < 1.25, and 0.75 < Bf < 1.25 [21].

#### 2.5.4. Optimization Procedure

The optimization procedure was performed to obtain the optimal levels of factors leading to the desirable responses. In order to define the workable optimum conditions, the model equations were graphically computed to visualize, by means of contour plots, the relationship between the factors and responses. Optimization with multiple responses was achieved using the desirability function. The experimental and predicted values of the responses were compared using a *t*-test in order to validate the adequacy of response surface models for predicting the optimum responses.

### 2.6. Sensory Analysis of Biscuits

Twenty-four hours following the production of biscuits, a sensorial evaluation was carried out. A total of 8 (3 men and 5 women) trained panelists were recruited from the staff of the Institute of Agricultural Research for Development (IRAD), Yaoundé, Cameroon. All panelists were volunteers and signed an informed consent sheet. A six-point scale was used to evaluate the biscuits from “0 = very low” to “5 = very high”. Panelists were recruited based on their regular consumption frequency of biscuits and their nonallergy to any foods. The panelists were asked to describe the intensity of the following descriptors: the flavor, hardness, granular, crispness, and overall quality of the biscuits. Samples were identified with three-digit code numbers and presented in a random sequence to the panelists. Water was provided to rinse the mouth between evaluations.

### 2.7. Nutritional Analysis

The nutritional composition of the optimized biscuit was assessed. The water content of the biscuit was determined by the gravimetric method [22]. The lipid content was assessed through extraction with petroleum ether as solvent [23] and the total nitrogen by the Kjeldahl method using 6.25 as the nitrogen conversion factor to total protein. Carbohydrates were determined by the Anthrone method [24], while dietary fibers were determined according to the method of Garbelotti et al. [25]. The energy values were calculated theoretically using the following conversion factors: 4.0, 4.0, and 9.0 kcal/g for proteins, carbohydrates, and fats, respectively [26]. The mineral profile of the biscuits (P, K, Ca, Mg, Na, Cu, Fe, Mn, and Zn) was determined by atomic absorption spectrophotometry (Varian Vista, Victoria, Australia).

### 2.8. Statistical Analysis

All experiments were performed in triplicates and values were expressed as means ± standard deviation. The data obtained was subjected to analysis of variance and regression analysis using Minitab software version 18.1 (Minitab Inc., USA). Graphs were plotted using SigmaPlot 12.5 version 12.5.0.38 (SYSTAT Software Inc., Chicago, IL, USA). The Student *t*-test was used to compare values and the statistical significance was set at *p* < 0.05.

## 3. Results and Discussion

### 3.1. Optimization of Biscuit Preparation

Table 2 presents the experimental matrix of the Doehlert design including the different combination of independent variables and the values of the responses assessed.

#### 3.1.1. Model Fitting

The results presented in Table 2 were exploited by applying the least-squares method and models were established. The following second-order polynomial equations explain the different responses measured as a function of the quantity of wheat flour, rice flour, margarine, and safou powder.

(2) $$\begin{array}{c} Y_{1}=1.148+0.00021X_{1}+0.01144X_{2}-0.00003X_{3}+0.00102X_{4}+0.00000X_{1}^{2}-0.00001X_{2}^{2}+0.00000X_{3}^{2}-0.00000X_{4}^{2}-0.00000X_{1}X_{2}+0.00000X_{1}X_{3}+0.000000X_{1}X_{4}-0.00000X_{2}X_{3}-0.00000X_{2}X_{4}-0.00000X_{3}X_{4},\\ Y_{2}=2.108+0.00140X_{1}+0.00806X_{2}-0.01133X_{3}+0.00025{\text{X}}_{4}+0.00000X_{1}^{2}-0.00000X_{2}^{2}+0.00001X_{3}^{2}+0.00000X_{4}^{2}-0.00000X_{1}X_{2}+0.00000X_{1}X_{3}-0.000000X_{1}X_{4}-0.00000X_{2}X_{3}-0.00001X_{2}X_{4}-0.00000X_{3}X_{4},\\ Y_{3}=3.636+0.00058X_{1}+0.00426X_{2}-0.00378X_{3}-0.00512X_{4}-0.00000X_{1}^{2}-0.00000X_{2}^{2}+0.00000X_{3}^{2}+0.00000X_{4}^{2}-0.00000X_{1}X_{2}+0.00000X_{1}X_{3}+0.000000X_{1}X_{4}-0.00000X_{2}X_{3}-0.00000X_{2}X_{4}-0.00001X_{3}X_{4},\\ Y_{4}=0.145+0.003166X_{1}+0.009644X_{2}-0.003980X_{3}+0.005310X_{4}-0.000002X_{1}^{2}-0.000004X_{2}^{2}+0.000002X_{3}^{2}-0.000003X_{4}^{2}-0.000007X_{1}X_{2}+0.000005{\text{X}}_{1}X_{3}+0.000001X_{1}X_{4}+0.000002X_{2}X_{3}-0.000005X_{2}X_{4}+0.000000X_{3}X_{4},\\ Y_{5}=5.917-0.00016X_{1}+0.00192X_{2}-0.00632X_{3}-0.01347X_{4}+0.00000X_{1}^{2}-0.00000X_{2}^{2}+0.00001X_{3}^{2}+0.00001X_{4}^{2}-0.00000X_{1}X_{2}-0.00000X_{1}X_{3}+0.000000X_{1}X_{4}+0.00000X_{2}X_{3}+0.00000X_{2}X_{4}+0.00001X_{3}X_{4}, \end{array}$$

where *Y*_1_ = crispness, *Y*_2_ = hardness, *Y*_3_ = aroma, *Y*_4_ = granulometry, *Y*_5_ = overall quality, *X*_1_ = wheat flour, *X*_2_ = rice flour, *X*_3_ = margarine, and *X*_4_ = safou powder.

The adequacy of these models to explain the variability in the different responses was evaluated by the coefficient of determination (*R*^2^), the adjusted *R*^2^, the absolute analysis of average deviation (AAD), the bias factor (Bf), the accuracy factor (Af), and the lack of fit.

Table 3 presents the values of the validation parameters. The second-order polynomial mathematical models were considered as valid taking into consideration the ranges recommended by Bas and Boyaci [21].

#### 3.1.2. Effects of Independent Variables on the Granulometry of Biscuits

**Figure 2:**
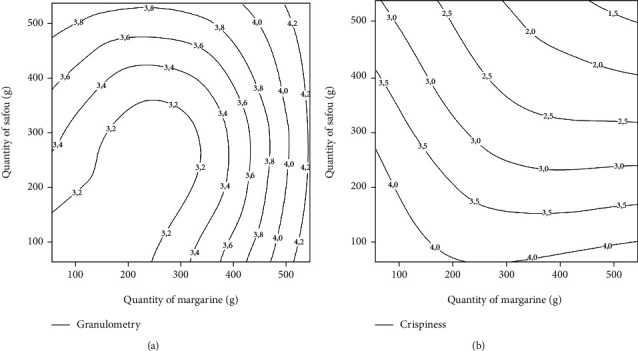
Effect of the quantities of margarine and safou powder on the granulometry (a) and the crispness (b) of safou rice biscuits.

Granulometry refers to the particle size which can be defined as a geometric property of texture linked to the perception of the size, shape, and number of particles of a product [27]. Different rice biscuits supplemented with safou powder as substitute of margarine were submitted to a panel for the evaluation of their granulometry. The contour plot (Figure 2(a)) shows simultaneous effect of the quantity of safou powder and margarine on the granulometry of the biscuits. The particle size of the biscuits increases following the addition of safou and margarine. This fact may indicate that the percentage of coarse fractions is what mostly affects this property. This could be explained by the fact that the finer fractions present similar particle sizes while the larger fractions have greater differences between them. Thus, a coarser particle size as obtained for composite flours is desirable for making hard dough biscuits [28].

#### 3.1.3. Effects of Independent Variables on the Crispness of Biscuits

Crispness of biscuits is one of the most desirable textural properties by consumers [29]. Crispness refers to the force required to break the biscuits and the sound emitted during that process. It was used in this study to assess the effect of substituting margarine with safou powder on the quality of rice biscuits. The sensory score for crispness ranged from 0 to 4.22 (Table 2).  Figure 2(b) shows the evolution of the crispness of the biscuits as a function of the quantity of safou and margarine. An increase in the amount of safou powder and margarine results in a reduction of crispness. This could be due to an increase in the lipid content of biscuits. In fact, safou powder has a lipid content of 61.56 g/100 g DM [14], and when added to those brought by margarine, it contributes to a rise in the lipid content of the biscuits and thus increases its softness [30]. The soft biscuits obtained lose their crispness. A similar observation was reported by Chakrabarti et al. [31]. They pointed out that addition of soybean, leguminous seeds containing high levels of lipids, contribute to the decrease the crispness of the biscuits obtained.

#### 3.1.4. Effects of Independent Variables on the Hardness of Biscuits

**Figure 3:**
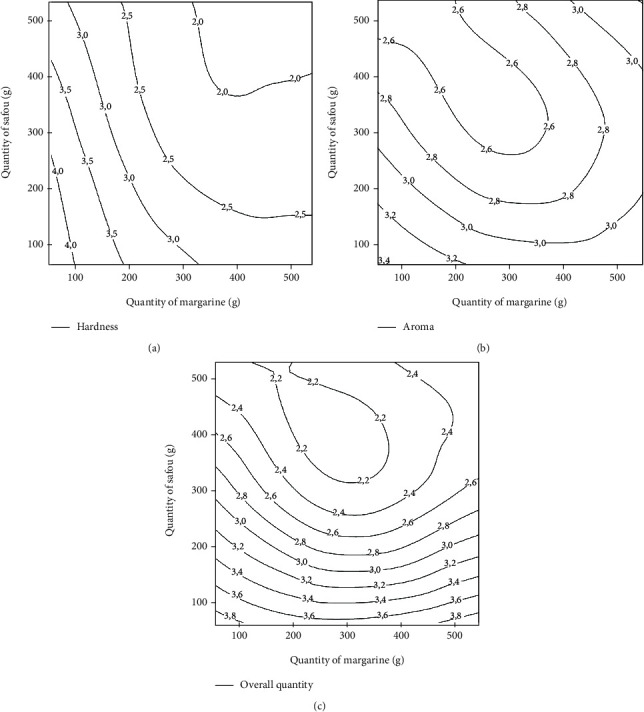
Effect of the quantity of margarine and safou powder on the hardness (a), aroma (b), and overall quality (c) of safou rice biscuits.

Hardness refers to the force required to compress a substance when placed between molar teeth [32]. The effect of safou powder and margarine on the hardness of biscuits is shown in Figure 3(a). An increase in the proportion of safou powder leads to an increase in the hardness of the biscuits. This could be explained by the fact that safou powder contains high amount of proteins 9.7–13.8 g/100 g DM [16] and fibers 9.91–13.95 g/100 g DM [17]. Hence, the water holding capacity of proteins and fibers brought by the safou powder will lead to a hard dough and thus to hard biscuits. Moreover, the high-water holding capacity of safou powder (724%) as shown by Eyenga et al. [14] could render the dough tough to knead and therefore increase the hardness of biscuits. The incorporation of *Dolichos lablab* powder with high water holding capacity in dough preparation also led to a significant increase of the cookie's hardness [33]. However, the contrary was observed with margarine, as an increase in its quantity led to a reduction in the hardness of the biscuits. This observation could be ascribed to the fact that in the presence of fats, flour is lubricated and the networks between the compounds responsible for the hardness of the biscuit are broken. Hence, the hardness gradually decreases leading to a soft biscuit, with an increased level of margarine [34]. Chakrabarti et al. [31] also reported that fats are generally used in biscuit preparation to soften and tenderize its texture.

#### 3.1.5. Effects of Independent Variables on the Aroma of Biscuits

Aroma is an important criterion that is used to assess the quality and desirability of a food product [35].  Figure 3(b) shows that when the quantity of safou powder increases, the characteristic aroma of the biscuit is not perceptible. In fact, safou is rich in aromatic compounds which have specific aroma. Thus, the aroma developed during baking through the Maillard reaction could be masked with aroma of the volatile compounds of safou as its proportion increases. Likewise, at high temperature, other molecules present in safou powder could interact with carbohydrates and result in the formation of a specific aromatic compound different from the characteristic one of biscuits. A similar observation was reported by Yildiz et al. [36]. The author concluded that when flours are heated, the interaction of their constituents can produce a desirable or undesirable aroma in the baked product. Another explanation could be the fact that ingredients used in biscuit formulation might confer its aroma to the final product. Pasqualone et al. [5] noticed a leafy aroma in biscuits made with almond skins. That aroma was characteristic of dried almond skins. Pasqualone et al. [37] reported an aroma typical of bran in biscuits formulated with acorns, aroma which was not found in the control one's free of acorn flour. Besides, an increase in the aroma of the cookies with the amount of margarine was noted. This underlines the important contribution of margarine in the development of the aroma of cookies.

#### 3.1.6. Effects of Independent Variables on the Overall Acceptability of Biscuits

The overall quality of the cookies is reduced when the quantity of safou powder increases (Figure 3(c)). This could be explained by the impact of safou powder on the determining factors of the product acceptability, especially its aroma. However, an increase in the quantity of margarine improves the overall quality of the cookies. This could be the fact that fats are the principal ingredients responsible for the tenderness of cookies and the enhancement of its texture, leading to an improvement of the quality of cookies [30]. According to Warinporn and Geoffrey [38], fats significantly contribute to improving the flavor of the finished product and thus increase its acceptability.

#### 3.1.7. Validation of Models

The optimum conditions for formulation of the dough that maximize the different responses used to assess the quality of rice biscuits were determined based on contour plots.  Table 4 presents the ranges for the amount of safou powder and margarine for which the sensory parameters of the biscuits were optimal. The biscuits had an optimum granulometry (4.20) when the quantity of safou powder varies from 420 to 600 g and the quantity of margarine from 320 to 600 g. The crispness of biscuits was optimal for the quantities of safou between 100 and 300 g and margarine between 320 and 600 g. The optimal hardness of biscuits (4.0) was recorded with safou between 100 and 550 g and margarine between 200 and 350 g. Regarding aroma, it was optimal (3.40) for safou values between 0 and 275 g and 0 and 500 g for margarine. Finally, the overall quality was optimal for the safou quantity ranging from 0 to 275 g and margarine 0 to 600 g.

In order to determine the conditions which satisfy all the responses assessed, a multiresponse optimization process was used through a desirability function. The optimum point was the one for which the desirability is closer to 1. That point was 20.24% wheat concentration, 24.51% rice, 19.09% margarine, and 2.47% safou powder. At this point, the biscuit produced had a granulometry of 3.53, aroma of 3.42, hardness of 2.70, crispness 3.99, and overall quality 4.02. (Table 5).

The adequacy of the model equations for predicting the response values was tested by comparing the experimental and predicted values at optimum conditions. The results showed that there is no significant difference (*p* > 0.05) between the predicted and the experimental values as observed in Table 5. This closeness between the experimental and predicted values under the optimum region confirmed the adequacy and suitability of the models.

### 3.2. Nutritional Characterization of the Optimized Biscuits

**Figure 4:**
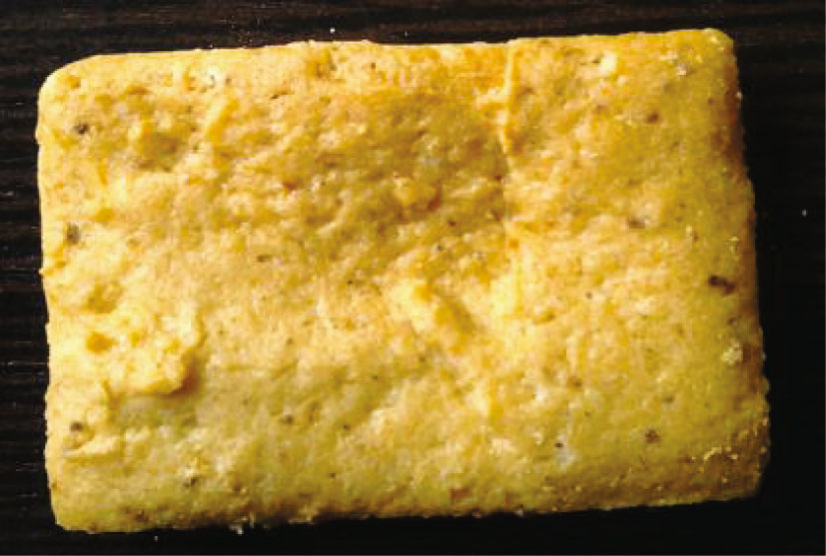
Optimized rice biscuit supplemented with safou.

The proximal composition of optimized biscuit illustrated in Figure 4 was determined. The moisture content of the biscuit was 4.45 ± 0.00% (Table 6). That moisture value is different to those reported for biscuits fortified with sesame seeds [39]. An explanation could be the great variability of the moisture according to the type of biscuits as highlighted by Akubor and Ukwuru [40]. The low moisture of the biscuit observed in this study suggests its good storage ability and a long shelf life. Sanni et al. [41] also reported that food products with low moisture have a long shelf life. As observed in Table 5, the protein content of the optimized biscuit was 10.13 ± 0.80 g/100 g DM. The value of the protein content observed in this study is higher than that obtained by Adeola and Ohizua [2] on biscuits made with flour of unripe cooking bananas, pigeon pea, and sweet potato in the proportion of 10 : 80 : 10. Hence, it showed the suitability of the biscuit made in this study to be used in the management of malnutrition. The total lipid content of the biscuit was 25.38 ± 0.02 g/100 g DM. This value appears high for such kind of food. However, it is important to highlight that a proportion of this fat was brought by safou used as substitute of margarine. Igile et al. [42] reported that the total unsaturated fatty acids of several varieties of safou varied between 50.00 and 69.48%. The authors highlighted linoleic acid as the most abundant fatty acid (34–47%) independently of the safou variety. Leudeu-Tchankou et al. [15] noticed that *D. edulis* fruit powder is rich in unsaturated fatty acid and contained 29.31% of oleic acid. Knowing the health benefits of unsaturated fatty acid acids, the biscuits prepared in this study appears interesting as they can be used for the management of cardiovascular diseases. The carbohydrate content of 55.1 ± 0.56 g/100 g DM found in the biscuit suggests their highly energetic potential. Hence, the energy density of the biscuit was calculated. The results showed that the optimized biscuit produced in this study has an energy density of 506.00 ± 0.70 kcal/g and could therefore be suitable for the management of malnutrition. Crude fibers were present in the optimized biscuit (2.56 ± 0.21 g/100 g DM). The presence of crude fibers in the biscuit suggests their therapeutic potential as fibers are useful in the prevention of heart diseases, colon cancer, digestive disorders, and diabetes [43]. The ash content of the biscuit was 3.55 ± 0.07 g/100 g DM. This value is higher than that of biscuit made from mushroom-wheat composite flours [44]. The enhancement of the ash content of biscuit might be attributed to the supplementation with safou powder. In fact, safou powder is a great source of minerals as reported in the literature by Kinkéla et al. [12]. The authors found an ash content of 5% in safou powder.

Globally, the nutritional value of the optimized biscuit was improved. The improvement observed could be attributed to safou powder used as substitute of margarine. In studies conducted by Ene-Obong et al. [13] and Eyenga et al. [14], the authors demonstrated that the nutritional values of biscuits supplemented with safou powder were significantly increased.

The mineral profile of the optimized biscuit was determined using atomic absorption spectrophotometry (Table 7). Ca, Mg, K, Na, and P were detected at concentrations of 0.035, 0.034, 0.192, 0.165, and 0.123 mg/100 g, respectively. Oligoelements such Zn, Cu, Mn, and Fe were found in the biscuit at concentrations of 34.8, 2.3, 4.8, and 33.60 ppm, respectively. These values are close to those reported by Eyenga et al. [14] with biscuits containing safou powder. Sodium and potassium play an important role in the human body. They are required to maintain osmotic balance of the body fluids and the pH of the body as well as to enhance normal retention of protein during growth. They also play an important role in the regulation of muscle and nerve irritability and in the control glucose absorption [44]. Iron is a major component of hemoglobin that carries oxygen to all parts of the body. It also has a critical role within cells assisting in oxygen utilization, enzymatic systems, especially for neural development, and overall cell function [45]. Magnesium, the cofactor of several enzymatic reactions, is essential for all living cells and zinc as an integral part of least 20 enzymes that belong to a large group known as metalloenzymes [46]. The presence of these minerals in the optimized biscuit suggests their potential health benefits.

The required daily intake (RDI) values for minerals in foods intended to human consumption were established by the United State Department of Agriculture [47]. For K, the RDI ranges from 0.4 to 0.8 g/day for infants, 2 to 2.3 g/day for adolescents, and 2.6 to 3.4 g/day for adults. Fe RDI is 0.00027 g/day for infants younger than 6 months and between 0.007 and 0.018 g/day for others. The RDI of Zn is 0.002–0.008 g/day for infants and children and 0.011–0.013 g/day for adolescent and adults. With regard to Mn, the RDI is 0.0003–0.001 g/day for infants, 0.001–0.003 g/day for children and adolescents, and 0.002–0.005 g/day for adults. The results obtained in this study suggest that the consumption of rice biscuits supplemented with safou will significantly contribute for improving the RDI of the population independently of their sex and age.

## 4. Conclusion

In this study, a new product made with wheat and rice flours, margarine, and safou powder was developed using response surface methodology. The optimum composition of dough leading to a biscuit with the most appreciated granulometry, crispness, hardness, aroma, and overall quality was 20.24% wheat flour, 24.51% rice, 19.09% margarine, and 2.47% safou powder. The physicochemical analyses of the optimized biscuit revealed its richness in nutrients and its high energy density. The results of this study demonstrated the suitability of safou as substitute of margarine in the production of rice and suggests the potential of the formulated biscuits in the management of malnutrition and noncommunicable diseases such as hypertension, obesity, and cardiovascular diseases.

## Figures and Tables

**Table 1:** Nutritional composition of rice flour and safou powder [14].

Components (g/100 g DM)	Matrix	
	Rice flour	Safou powder
Water content	9.30	4.77
Proteins	7.10	8.23
Lipids	0.50	61.56
Sugars	81.10	23.53
Fiber	1.40	1.90
Ash	0.60	2.55

DM: dry matter.

**Table 2:** Design of the experiment and responses of the Doehlert design for the production of rice biscuits supplemented with safou powder.

Run	Wheat	Rice	Margarine	Safou	Granular	Crispness	Hardness	Aroma	Overall quality
1	0.00	600.00	300.00	300.00	4.06	3.25	2.18	3.43	2.75
2	1200.00	600.00	300.00	300.00	2.93	4.12	3.68	2.31	2.37
3	300.00	80.38	300.00	300.00	3.31	2.75	2.31	3.43	3.18
4	900.00	1119.61	300.00	300.00	0.00	0.00	0.00	0.00	0.00
5	300.00	1119.61	300.00	300.00	3.75	3.10	2.55	2.65	2.35
6	900.00	80.38	300.00	300.00	3.77	2.50	2.66	3.05	2.55
7	300.00	426.79	55.05	300.00	3.83	3.72	3.61	3.00	2.80
8	900.00	773.20	544.94	300.00	4.22	2.66	2.50	2.72	2.66
9	300.00	773.20	544.94	300.00	4.25	2.30	1.80	2.79	2.83
10	900.00	426.79	55.05	300.00	3.66	4.11	4.16	3.27	3.27
11	600.00	253.58	544.94	300.00	4.16	2.83	2.44	3.33	2.55
12	600.00	946.41	55.05	300.00	2.88	3.88	3.94	2.57	2.77
13	300.00	426.79	238.76	62.82	2.55	4.00	2.27	3.33	3.80
14	900.00	773.20	361.23	537.17	3.94	1.66	1.88	2.72	2.61
15	300.00	773.20	361.23	537.17	4.00	1.44	1.66	2.72	2.16
16	900.00	426.79	238.76	62.82	3.22	4.11	3.88	3.43	3.92
17	600.00	253.58	361.23	537.17	3.77	2.16	2.05	3.27	2.33
18	600.00	946.41	238.76	62.82	3.77	3.88	3.72	2.77	3.08
19	600.00	600.00	116.28	537.17	3.88	2.75	2.83	2.50	2.20
20	600.00	600.00	483.71	62.82	4.05	4.22	2.72	3.11	3.83
21	600.00	600.00	300.00	300.00	4.05	3.50	2.44	2.94	2.50

**(a):** 

Factors	Crispness				Hardness				Aroma			
	DL	Coef	*F*	*p*	DL	Coef	*F*	*p*	DL	Coef	*F*	*p*
*X*_0_: constant		1.14896				2.10825				3.63602		
*Linear*												
*X*_1_: wheat	1	0.00021	0.01	0.907	1	0.00140	0.61	0.440	1	0.00058	0.19	0.667
*X*_2_: rice	1	0.01144	29.41	0.000^∗^	1	0.00806	14.73	0.001^∗^	1	0.00426	7.31	0.012^∗^
*X*_3_: margarine	1	−0.00003	0.00	0.995	1	−0.01133	7.36	0.011^∗^	1	−0.00378	1.46	0.238
*X*_4_: safou	1	0.00102	0.07	0.801	1	0.00025	0.00	0.950	1	−0.00512	2.92	0.099
*Quadratic*												
*X*_1_^2^	1	0.00000	0.18	0.677	1	0.00000	1.24	0.275	1	−0.00000	0.04	0.836
*X*_2_^2^	1	−0.00001	19.16	0.000^∗^	1	−0.00000	4.27	0.049^∗^	1	−0.00000	6.64	0.016^∗^
*X*_3_^2^	1	0.00000	0.03	0.872	1	0.00001	6.02	0.021^∗^	1	0.00000	0.55	0.464
*X*_4_^2^	1	−0.00000	0.95	0.338	1	0.00000	0.17	0.683	1	0.00000	0.41	0.528
*Interaction*												
*X*_1_ *X*_2_	1	−0.00000	15.41	0.001^∗^	1	−0.00000	16.16	0.000^∗^	1	−0.00000	3.85	0.528
*X*_1_ *X*_3_	1	0.00000	1.47	0.236	1	0.00000	2.13	0.156	1	0.00000	17.52	0.000∗
*X*_1_ *X*_4_	1	0.00000	1.16	0.291	1	−0.00000	0.83	0.370	1	0.00000	0.52	0.479
*X*_2_ *X*_3_	1	−0.00001	4.39	0.046^∗^	1	−0.00000	2.55	0.122	1	−0.00000	0.93	0.344
*X*_2_ *X*_4_	1	−0.00000	3.05	0.092	1	−0.00001	4.18	0.051	1	−0.00000	0.23	0.633
*X*_3_ *X*_4_	1	−0.00001	0.87	0.360	1	0.00000	0.53	0.473	1	0.00000	4.06	0.054
Pure error	21				21				21			
Lack of fit	6	1.18559			6	1.17415			6	0.66054		
Total	41				41				41			
*R* ^2^		84.89%				81.39%				81.48%		
Adj-*R*^2^		77.06%				71.73%				71.88%		
AADM		0.24				0.43				0.25		
Af		1.18				1.19				1.20		
Bf		1.15				1.09				1.06		

DF: degrees of freedom; ^∗^significant at *p* < 0.05.

**(b):** 

Factors	Granulometry					Overall quality		
	DL	Coef	*F*	*p*	DL	Coef	*F*	*p*
*X*_0_: constant		0.145111				5.91773		
*Linear*								
X_1_: wheat	1	0.003166	2.74	0.109	1	−0.00016	0.01	0.922
*X*_2_: rice	1	0.009644	18.43	0.00^∗^	1	0.00192	1.09	0.306
*X*_3_: margarine	1	−0.00398	0.79	0.381	1	−0.00632	2.97	0.096
*X*_4_: safou	1	0.00531	1.54	0.225	1	−0.01347	14.74	0.001^∗^
*Quadratic*								
*X*_1_^2^	1	−0.000002	1.38	0.251	1	0	0.03	0.873
*X*_2_^2^	1	−0.000004	11.55	0.002^∗^	1	0	2.85	0.103
*X*_3_^2^	1	0.000002	0.22	0.644	1	0.00001	2.82	0.105
*X*_4_^2^	1	−0.000003	0.32	0.576	1	0.00001	4.9	0.035^∗^
*Interaction*								
*X*_1_ *X*_2_	1	−0.000007	29.73	0.00^∗^	1	0	7.33	0.012^∗^
*X*_1_ *X*_3_	1	0.000005	3.59	0.069	1	0	0	0.959
*X*_1_ *X*_4_	1	0.000001	0.13	0.72	1	0	2.53	0.123
*X*_2_ *X*_3_	1	0.000002	0.33	0.572	1	0	0.04	0.849
*X*_2_ *X*_4_	1	0.000005	3.47	0.074	1	0	0.81	0.377
*X*_3_ *X*_4_	1	0	0	0.985	1	0.00001	1.26	0.272
Pure error	21				21			
Lack of fit	6	1.34469			6	0.90616		
Total	41				41			
*R* ^2^		76.84%				79.75%		
Adj-*R*^2^		64.83%				69.25%		
AADM		0.57				0.4		
Af		1.21				1.18		
Bf		1.18				0.77		

DF: degrees of freedom; ^∗^significant at *p* < 0.05.

**Table 4:** Optimal conditions of biscuits production.

Responses	Independent variables	
	Safou (g)	Margarine (g)
Granulometry	420–600	320–600
Crispness	100–300	320–600
Hardness	100–550	200–350
Aroma	0–275	0–500
Overall quality	0–275	0–600

**Table 5:** Predicted and experimental values of the responses obtained at optimum conditions.

Responses	Predicted values	Experimental values	*p* value (*t*-test)
Granular	3.53	3.50 ± 0.03	0.143
Crispness	3.99	3.93 ± 0.06	0.274
Hardness	2.7	2.65 ± 0.07	0.561
Aroma	3.43	3.40 ± 0.20	0.806
Overall quality	4.02	4.16 ± 0.16	0.559

**Table 6:** Proximate composition of the optimized biscuit.

Parameters	Values
Moisture (%)	4.45 ± 0.00
Fats (g/100 g DM)	25.38 ± 0.02
Sugars (g/100 g DM)	55.10 ± 0.56
Proteins (g/100 g DM)	10.13 ± 0.80
Ash (g/100 g DM)	3.55 ± 0.07
Crude fibers (g/100 g DM)	2.56 ± 0.21
Energy (kcal/100 g)	506.00 ± 0.7

**Table 7:** Mineral composition of the optimized biscuit.

Minerals	Values
Ca (mg/100 g)	0.035
Mg (mg/100 g)	0.034
K (mg/100 g)	0.192
Na (mg/100 g)	0.165
P (mg/100 g)	0.123
Zn (ppm)	34.81
Cu (ppm)	2.3
Mn (ppm)	4.8
Fe (ppm)	33.60

## Data Availability

Upon request, the data used in this study are available from the corresponding author.
